# Molecular phylogeny reveals *Varroa* mites are not a separate family but a subfamily of Laelapidae

**DOI:** 10.1038/s41598-024-63991-z

**Published:** 2024-06-18

**Authors:** Jaeseok Oh, Seunghyun Lee, Woochan Kwon, Omid Joharchi, Sora Kim, Seunghwan Lee

**Affiliations:** 1 Insect Biosystematics Laboratory, Department of Agricultural Biotechnology, Seoul National University, 1, Gwanak-ro, Gwanak-gu, Seoul, Republic of Korea; 2 Research Institute of Agriculture and Life Sciences, Seoul National University, Seoul, Republic of Korea; 3 Department of Life Sciences, Natural History Museum, London, UK; 4 Division of Environmental Science and Ecological Engineering, Korea University, Seoul, South Korea; 5 Anatis Bioprotection Inc., Saint-Jacques-de-Mineur, Québec, J0J 1Z0 Canada; 6 All-Russian Institute of Plant Protections, St. Petersburg, Russia; 7 Agriculture Science and Technology Institute, Andong National University, Andong, Republic of Korea; 8 Lab. of Insect Phylogenetics and Evolution, Department of Plant Protection & Quarantine, Jeonbuk National University, Jeonju, 54896 Republic of Korea; 9 Department of Agricultural Convergence Technology, Jeonbuk National University, Jeonju, 54896 Republic of Korea

**Keywords:** Multi-locus phylogeny, Mitochondrial genome, Varroinae, Laelapidae, Taxonomic change, Host evolution, Evolution, Zoology

## Abstract

*Varroa* mites, notorious for parasitizing honeybees, are generally classified as Varroidae. Their extremely modified morphologies and behaviors have led to debates regarding their phylogenetic position and classification as an independent family. In this study, two different datasets were employed to reconstruct the phylogenies of *Varroa* mites and related Laelapidae species: (1) 9257 bp from the whole 13 mitochondrial protein-coding genes of 24 taxa, (2) 3158 bp from 113 taxa using Sanger sequencing of four nuclear loci. Both mitochondrial and nuclear analyses consistently place *Varroa* mites within the Laelapidae. Here we propose to place *Varroa* mites in the subfamily Varroinae stat. nov., which represents a highly morphologically adapted group within the Laelapidae. Ancestral state reconstructions reveal that bee-associated lifestyles evolved independently at least three times within Laelapidae, with most phoretic traits originating from free-living ancestors. Our revised classification and evolutionary analyses will provide new insight into understanding the *Varroa* mites.

## Introduction

*Varroa* mites, represented by the notorious *Varroa destructor* Anderson and Trueman, 2000, are one of the most well-known honeybee pests in the world^1,2^. *Varroa* mites are defined as a family, Varroidae, consisting of six species in two genera^3^: *Varroa jacobsoni* Oudemans; *Varroa underwoodi* Delfinado-Baker and Aggarwal; *Varroa rindereri* Guzman and Delfinado-Baker; *Varroa destructor* Anderson and Trueman; *Euvarra sinhai* Delfinado and Baker; *Euvarroa wongsirii* Lekprayoon and Tangkanasing. All *Varroa* mites have remarkably adapted morphologically, physiologically, and ecologically to various species of honey bee^4–7^. Among them, *V. destructor* is the most widespread species in the group, posing a great threat to the overall apicultural and pollination industry after successfully adapting to the European honeybee (*Apis mellifera* Linnaeus) from its original host Asian honeybee (*Apis cerana* Fabricius)^8,9^. Currently, *V. destructor* is considered one of the major causes of colony collapse disorder (CCD) in European honeybees, serving as the primary vector for numerous contagious bee viruses such as Deformed Wing Virus (DWV)^10^. Likewise, the impact of *Varroa* mites extends beyond direct effects on bees and encompasses indirect consequences, including economic implications such as reduced pollinator populations, decreased crop yield, and increased expenditure on pest control^11,12^.

Despite numerous studies of *Varroa* mites, their phylogenetic relationship among related taxa remains largely unexplored. Currently, *Varroa* mites are widely accepted as a separate family, Varroidae, without much debate^1,2,13–16^ with only a few exceptions^17,18^. However, the classifications of *Varroa* mites had been the subject of prolonged controversy due to their unique external morphologies and parasitic life cycle. The genus *Varroa* was first established by Oudemans^19^ based on *V. jacobsoni* from the hive of *Apis cerana indica* Fabricius on Indonesian Java Island, by having both (i) broad ventral shields and (ii) reduced cheliceral digit. The genus *Varroa* was initially placed within Laelaptinae based on (i) metapodal and anal shield and (ii) numerous dorsal setae. Delfinado and Baker^4^ elevated *Varroa* to the family Varroidae based on densely dorsal setae, lack of fixed digit, looped stigma, and its unique leg-chaetotactic system (Supplementary Fig. 1). Casanueva^20^ asserted the tribe Varroini that belongs to the Hymenopteran-associated laelapid group, Melittiphidinae, by morphological phylogenetics using 83 characters. Casanueva^20^ suggested unique regressive apomorphies of *Varroa* also existed in some other laelapid genera, such as reduced gnathosomal setae in *Tropilaelaps*, *Stevelus*, *Dinogamasus*, and *Urozercon*, and reduced cheliceral digit in *Myrmolaelaps*. All the aforementioned morphological studies have in common that *Varroa* mites are closely related to Laelapidae but focused on whether to assign their taxonomic rank as a genus, tribe within Laelapidae, or a separate family.

Existing molecular phylogenies including *Varroa* mites, Laelapidae, and their relatives, have consistently indicated a close association between *Varroa* mites and Laelapidae, aligning with the findings from previous morphological analyses (Multi-locus phylogeny^21–23^; Mitogenome phylogeny^24–27^; Phylogenomics^28^). *Varroa* mites have been either placed within Laelapidae^22–24^, recovered as a sister group to Laelapidae^28^, or shown close affinity with Laelapidae + Blattisociidae^27^. However, most studies only included *V. destructor*^22–24,27^, utilized fewer genes below the current standard^21–23^, or lacked *Varroa*-related Laelapidae species in their taxon sampling, hindering the examination of whether *Varroa* mites constitute a separate family or are nested within other Laelapidae groups^24,27,28^. Therefore, an inclusive approach incorporating multiple *Varroa* mite species, broader coverage of Laelapid mites, and robust phylogenetic tree reconstruction with an extensive genetic dataset could elucidate their precise phylogenetic placement and aid in determining the most appropriate taxonomic rank for *Varroa* mites.

Another important aspect concerning the relationship between *Varroa* mites and Laelapidae pertains to the association between host use and phylogeny. *Varroa* mites are exclusively parasitic on bees, while Laelapidae exhibit a diverse array of parasitic behaviors. Previous studies have broadly categorized Laelapidae mites and their relatives based on their host types into three categories: (i) free-living, (ii) vertebrates, and (iii) arthropods^29–32^. Morphological analyses conducted by Casanueva^20^ revealed distinct monoclades sharing similar hosts among the included members, such as free-living forms, Hymenoptera parasites, beetles, and roach riders. Casanueva^20^ placed *Varroa* mites within Laelapidae, forming a monoclade alongside other bee-parasitic laelapids like *Dinogamasus*, *Tropilaelaps*, *Urozercon*, and *Stevelus*. However, Dowling and OConnor^22^ used two-loci multi-locus phylogenetics and identified multiple origins of both vertebrate and invertebrate parasitism. This contradicted Casanueva's findings, notably observing that *Dinogamasus* spp., a Carpenter bee parasite, located far apart from *Varroa destructor* on phylogeny. By employing a more reliable phylogenetic tree and a more refined host categorization than the existing ones, may unravel whether morphological traits of *Varroa* mites and Laelapidae are shaped by their hosts and how phoretic behavior aids in establishing a more robust classification.

In this study, we provide phylogenetic trees with the most up-to-date sample coverage that includes multiple species of *Varroa* mites and closely related groups such as Laelapidae, as well as other outgroup families. Based on our results, we were able to reach the Laelapidae *s.lat.* including Varroinae which is relegated from its family level. To confirm our phylogenetic hypothesis more robustly, we reconstructed phylogenetic trees using two different sampling strategies and types of molecular datasets widely employed in Acari phylogenies^22–24,26^: (i) mitochondrial genome sequences with a small number of terminal species and (ii) Sanger-based multi-locus data with a larger number of terminal species. We further utilized the resulting multigene phylogeny to re-examine the evolutionary history of the parasitic lifestyle within Laelapidae, an aspect that had not been extensively studied after Casanueva^20^ and Dowling and OConnor^23^. Our ancestral state reconstruction along with their host association suggests that the *Varroa* mite’s bee parasitic behavior evolved independently.

## Result

### Phylogenetic analysis of mitogenome sequences

**Figure 1:**
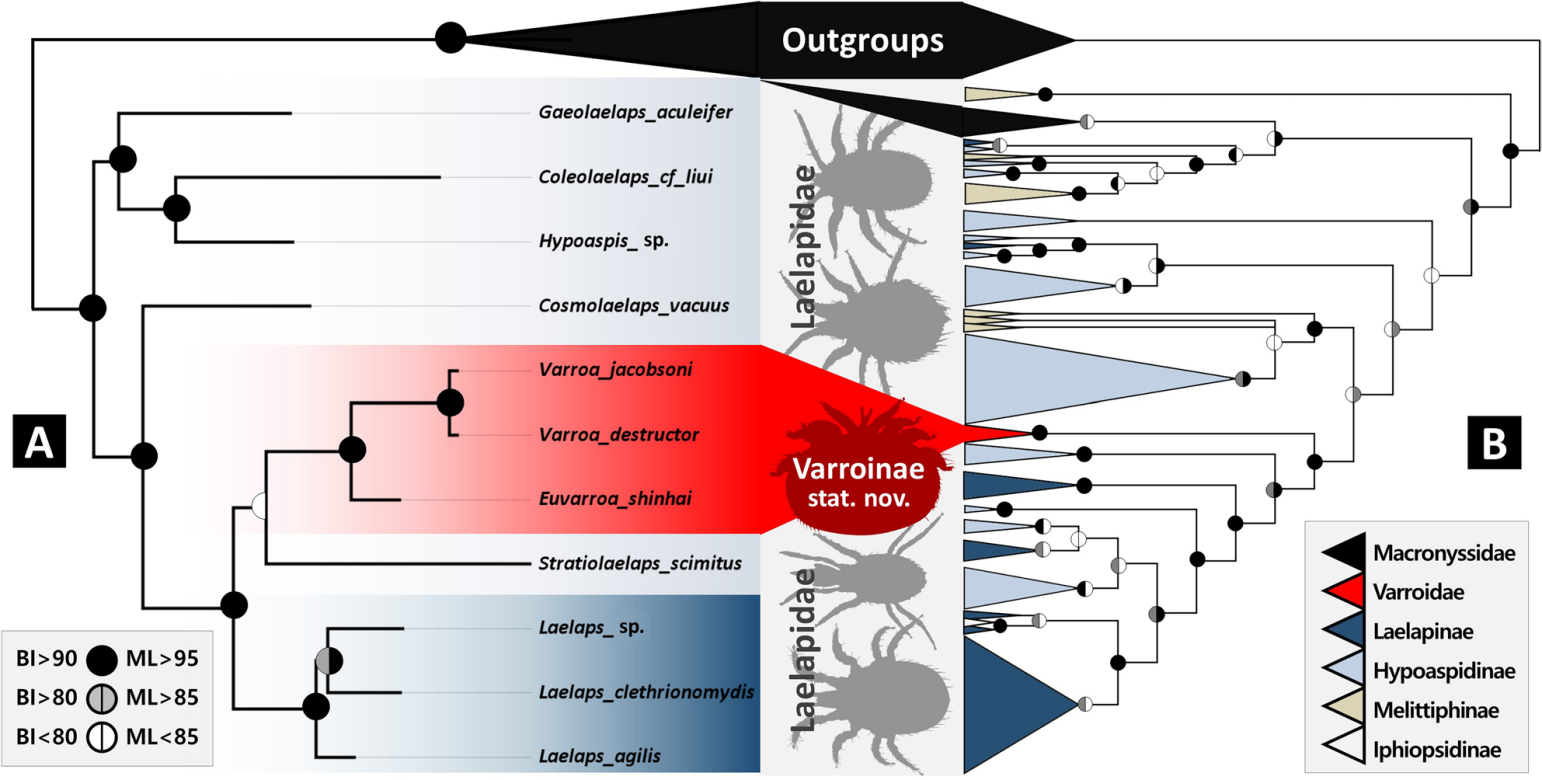
Combined phylogenetic tree of varroa mites and relatives. (**A**) Mitogenome phylogeny. (**B**) Multi-locus phylogeny. The tree shown are resulted from Bayesian inference. Node colors represent posterior probabilities from MrBayes and ultrafast bootstrap values from IQ-tree. Monophyletic clades in the multi-locus phylogeny are shown as triangles for better visualization. Refer to Fig. 2A for comprehensive node details.

A total of 9257 bp (atp6: 651 bp, atp8: 174 bp, cox1: 1470 bp, cox2: 665 bp, cox3: 753 bp, cytb: 1008 bp, nad1: 768 bp, nad2: 783 bp, nad3: 327 bp, nad4: 939 bp, nad4L: 225 bp, nad5: 1143 bp, and nad6: 351 bp) were obtained from a combined dataset. This dataset included only the family Laelapidae and Varroidae among the superfamily Dermanyssoidea Laelapidae *s.lat.* with strong support (UFB = 100/BS = 100) (Fig. 1). Those laelapid mites were found to be sister to the clade containing superfamily Parasitoidea and Celaenopsoidea (UFB = 77/BS = 98) in both Maximum Likelihood (ML) and Bayesian Inference (BI) analyses (Supplementary Figs. 2, 3). Although the topologies of the constructed trees obtained from ML and BI analyses were not always congruent, Varroidae consistently belonged to Laelapidae *s. lat.* in both analyses. Varroidae was supported as a monophyletic group (UFB = 100/BS = 100) among the 11 ingroup taxa, which consisted of two genera and three species, including its type species *V. jacobsoni*. The other ingroups comprised the two broadest Laelapid superfamilies, Hypoaspidinae and Laelapinae. Hypoaspidinae (*Coleolaelaps*, *Cosmolaelaps*, *Gaeolaelaps*, *Hypoaspis*, and *Stratiolaelaps*) also exhibited non-monophyletic relationships in both ML and BI analyses. Furthermore, the genus *Stratiolaelaps* formed a sister group relationship with the clade of *Laelaps* with low support (UFB = 51/BS = –), as observed in the multi-locus phylogeny. Varroidae was formed to sister to *Stratiolaelaps scimitus* Berlese with low support (UFB = –/BS = 56) in the BI analysis, but this was not observed in the ML analysis.

### Phylogenetic analysis of multi-locus sequences

The dataset, encompassing 3,158 base pairs (28S: 798 bp, 18S: 1557 bp, ITS: 431 bp, and H3: 372 bp), led to the generation of topologies through BI and ML methods. These topologies were mostly congruent, though there were some divergences at certain nodes (Supplementary Figs. 4, 5). Notably, *Varroa* mites were accurately positioned within the Laelapidae family, exhibiting a close affinity with clade A, which itself demonstrated strong support, encompassing numerous species (UFB = 95/BS = 96). The topology of *Varroa* mites, *Stratiolaelaps*, and *Laelaps* mirrored that found in the Bayesian mitogenome tree. Among the included families, only the Macronyssidae (UFB = 88/BS = 76) and Varroidae (UFB = 100/BS = 100) formed monophyletic groups with moderate to strong support. However, the subfamilies Hypoaspidinae, Laelapinae, and Melittiphinae did not demonstrate monophyly, exhibiting varying levels of support. Hypoaspidinae fragmented into 12 clades with mostly non-monophyletic genera, except for the strongly supported monophyletic *Stratiolaelaps* (UFB = 100/BS = 100). Laelapinae subdivided into six clades, with *Andreacarus* being the only genus forming a monophyletic relationship, supported by strong values (UFB = 100/BS = 100). Lastly, Melittiphinae was divided into four disparate clades, with *Gymnolaelaps* and *Tropilaelaps* forming well-supported monophyletic clades (UFB = 100/BS = 100).

### Evolution of Laelapidae phoretic traits

**Figure 2:**
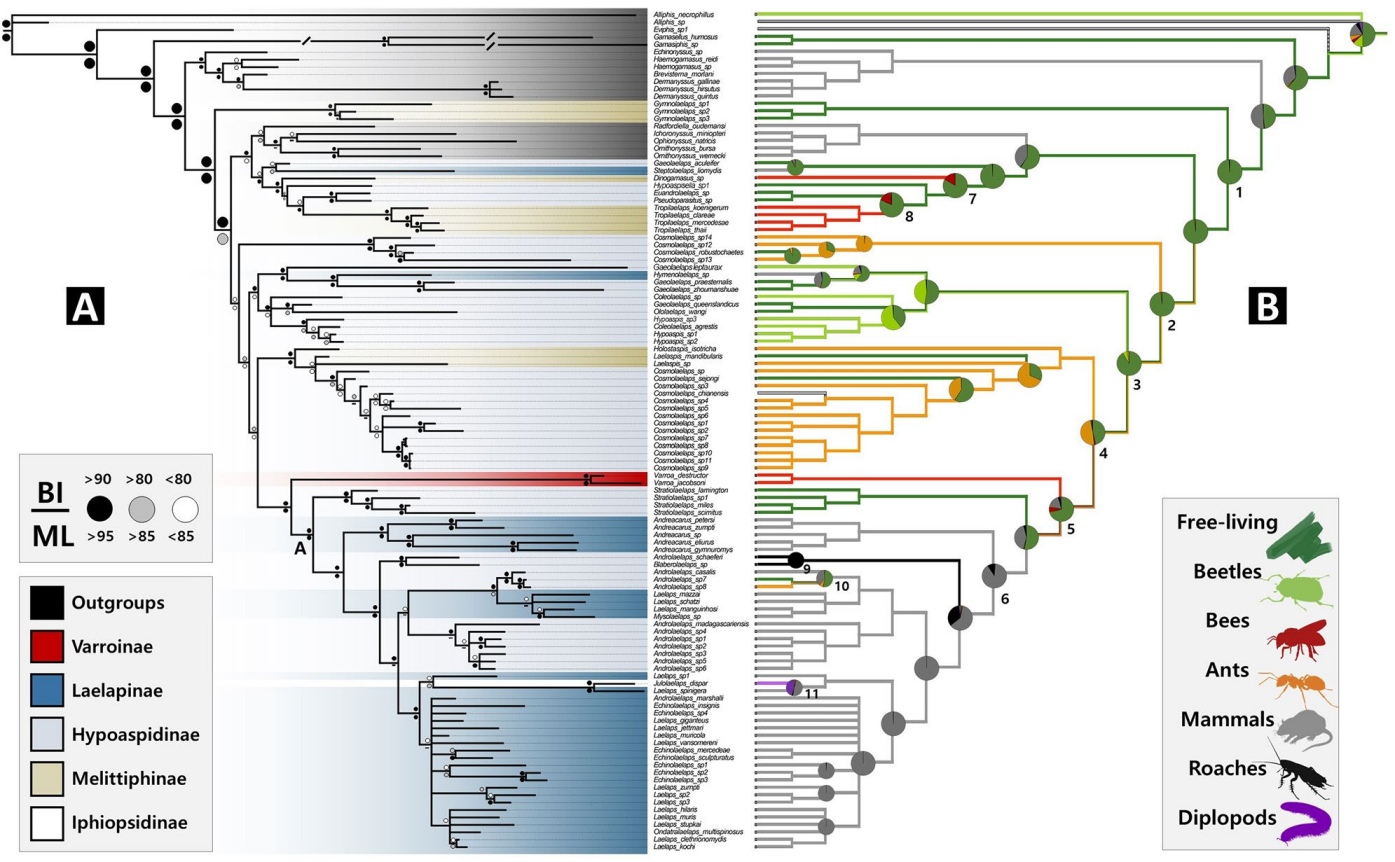
(**A**) Multi-locus phylogeny resulting from MrBayes. (**B**) Summary of Parsimony and Bayesian Ancestral State Reconstructions for Phoretic Hosts. Branch colors depict the results of ancestral state reconstruction via parsimony analysis. The pie charts display mean posterior probability values computed through RJ-MCMC analysis using BayesTraits.

The ancestral states of phoretic traits reconstructed by Mesquite and Bayestraits generally showed congruent results (Fig. 2). The most probable common ancestral trait within Laelapidae *s.lat.* was 'free-living', with an estimated probability of approximately 98% (node 1). This trait also appeared as the most probable ancestral trait at various deeper nodes on the trees (nodes 2, 3, 4). Phoretic behavior on ants and bees is estimated to have originated at least three times independently within Laelapidae *s.lat.*, all likely evolving from a free-living ancestry (ants: nodes 2, 4, 10; bees: nodes 7, 8, 5). Beetle phoretic behavior evolved at least twice within closely related clades from a free-living ancestry (node 3). The parasitic behavior of *Varroa* mites also appears to have evolved from a free-living ancestry. Reverse transitions from invertebrate hosts to free-living habits were seldom observed. Rodent parasites arose from at least three clades, with multiple terminal species within the subfamily. In the most species-rich rodent phoretic clade (node 6) of our phylogenetic tree, phoresy of roaches (node 9) and diploids (node 11) evolved at least once within the family from rodent phoresy.

## Discussion

Our study has revealed significant challenges in the current classification of Laelapidae. These include seriously non-monophyletic Laelapidae subfamilies and paraphyletic Laelapidae under multi-locus phylogenetics, when Macronyssidae is included. The monophyly of Laelapidae under mitochondrial genome phylogeny (Fig. 1) can be interpreted as a result of better and larger genetic data, but it may also be due to a simple sampling bias, with missing Macronyssidae in the sampling. Despite these challenges, it is clear that conventional morphology and molecular phylogenetic analyses are inconsistent, and a new perspective not bound by traditional morphology is needed to develop a more stable phylogeny. While our sampling of Laelapidae is limited and lacks many representative taxa, including type genera and species of each subfamily, and a more densely sampled multi-locus analysis showed low supports on the deeper nodes, we have not made significant changes to the taxonomic ranks. One major discovery of our study is that the *Varroa* mites have a close affinity to *Laelaps*, the type genus of the Laelapidae family. This was identified by both four molecular phylogenetic results, namely multi-locus and mitochondrial phylogenetic results, under both ML and BI frames (Fig. 1). This discovery reasonably relegates the disreputable *Varroa* mites, currently known as a family Varroidae, to a subfamily within Laelapidae. Based on our molecular analysis and prior morphological studies^31–39^, additional morphological evidence was identified regarding crucial characteristics that play a significant role in establishing the family Varroidae. (i) cheliceral structure: Oudemans^19^ noted the similarity of the chelicerae of female *Berlesia* to those of *Varroa* and their modifications for parasitism; he also noted that the involution of the cheliceral digit is dissimilar. Looking for comparisons with *Varroa* chelicerae in other parasitic Acari, Oudemans^19^ found similarities with those of *Berlesia* and ticks in terms of their construction for piercing the host cuticle, although *Varroa* lacks retrospective anchoring structures for attachment to its host as in the latter two taxa. In particular, a similar reduction in the size of the fixed digit was observed in the females of *Varroa* and *Berlesia*. The chelicerae of *Varroa* tend to have less robust movable digits with a vertical cutting plane typical of mesostigmatid mites, and the cutting actions of the right and left digits remain functionally independent^40^ and appear to be restricted to the tips of the cheliceral shafts. (ii) internal hypostomal setae (*h3*): The subcapitulum generally bears a pair of palp coxal setae, and the hypostome bears three pairs of setae (*h1*–*3*) arranged in a triangular pattern. The *h3* is lost in some genera of Laelapidae, such as *Dicrocheles* and some *Jacobsonia*^32^. (iii) hypertrichy of idiosoma: This character can be easily found in the family Laelapidae, as Oudemans^19^ rightly pointed out the similarity of *V. jacobsoni* with *Pogonolaelaps canestrinii* (mentioned as *H. canestrinii*) with regard to the hypertrichy of the dorsal shield. Also, in the genera such as *Eulaelaps*, *Haemogamasus*^41^, *Dinogamasus*, *Neohypoaspis*, *Sphaeroseius*, *Suracarus*, and *Tropilaelaps*, the hypertrichy character can be observed^32,38,42^. (iv) extra setae on genital shield: The genital shield generally only bears the *st5* setae. However, if the shield is posterolaterally expanded, it bears 1–5 additional setae pairs (*Jv1–3*, *Zv1–2*) or more in cases of hypertrichy, as in the genera *Eulaelaps*, *Haemogamasus*, or *Laelaps*^41^ or in the myrmecophilous genus *Sphaeroseius*^32^. (v) enlarged metapodal plates: The family Laelapidae generally have one or two pairs of small metapodal plates, but sometimes greatly enlarged, as in the genera *Eulaelaps*^41^, and *Neohypoaspis*^32^. (vi) conspicuously short legs: found in some arthropod-related genera such as *Conolaelaps*, *Dynatochela, Scorpionyssus*, and some *Myrmozercon* species. Their legs are shortened compared to the idiosoma. Additionally, according to Burkett-Cadena^43^, the most notable characteristics of parasitic arthropods are their body shape, mouthparts, and leg shape. These specifically modified features can be found in other parasitic arthropods such as Myrmecophilidae (ant cricket), Mystacinobiidae (bat fly), and other nest-dwelling mites like *Tropilaelaps*, *Melittiphis*, and *Myrmozercon*. These inquilines have undergone changes in their morphology to adapt to specialized environments. Thus, we can assume the modified appearance of *Varroa* mites is a result of the optimal adaption to the limited space of the bee hive, and Varroidae should be demoted and transferred to Varroinae stat.nov., with the specific unique morphological features previously assigned to Varroidae.

Furthermore, at the viewpoint of host traits, our findings revealed that parasitic traits tend to evolve from a free-living state to various invertebrates and vertebrates independently, with a few reverse transitions to invertebrates that share similar habitats with closely related mammal host species (Fig. 2). Similarly, the parasitic behavior observed in *Varroa* mites most likely evolved from a history of free-living (69%). However, a significant challenge arose at Node 5 due to insufficient support, rendering uncertain their origin from a free-living state. Minor alterations in topology significantly impact the reconstruction of ancestral states, leaving open the possibility that the evolution of bee parasitic behavior in *Varroa* mites might be linked to ant phoretic behavior. To address this, it is necessary to add more terminal species that are possibly related to *Varroa* mites (e.g., *Myrmozercon*) into our molecular tree or use markers with better resolution to enhance support across nodes. Our results indicate the host traits may play a significant role in the classification of the family Laelapidae, as host specificity is frequently observed in numerous groups within the subclass Acari^30,32,44–46^. In particular, Casanueva^20^, which, although not widely used today, seems to be reevaluated positively which integrated host and morphology in its classifications. We found that the highly supported clades tended to be in slightly better concordance with paraphyletic characters rather than traditional morphological classifications. Examples include the relegation of *Varroa* from an independent family to Laelapidae, and the formation of a highly supported monoclade (node 4) of Melittiphinae (*Laelaspis*, *Holostaspis*) and Hypoaspidinae (*Cosmolaelaps* partim) found in ants, Laelapinae (*Andreacarus*, *Laelaps*, *Mysolaelaps*, *Echinolaelaps*, *Ondatralaelaps*), Hypoaspidinae (*Blaberolaelaps*, *Androlaelaps*), and Ipiopsidinae (*Julolaelaps*), which primarily host rodents and some of the organisms that live around them, form a high-supporting monoclade. In this context, the genus *Androlaelaps* may be included in the subfamily Laelapinae a subfamily reliant on mammals (node 6), as their close affinity has been the subject of occasional debate in several prior studies^23,47^. Given the independent evolution of similar phoretic behaviors across diverse clades, a comprehensive analysis is required, extending beyond host associations, and encompassing morphological and phylogenetic analyses. For example, bee-associated behavior has independently evolved at least three times (node 5, 7, and 8) within Laelapidae *s.lat.*, paralleling the independent occurrences of ant phoretic behavior (node 2, 4), also observed at least three times. This independent evolution of traits is supported by highly robust MRCA nodes across nearly all clades. The initial step toward refining classification in line with phylogenetic relationships entails identifying the morphological characteristics at each node. In the future, we will need to revise not only Varroinae but also other subfamily classifications based on trees that include more terminal species, type species, and genera using more powerful markers, and in the process, we learned that the role of phoretic behavior should not be underestimated.

### Systematic account of Varroinae

Hyporder (Subcohort) Dermanyssiae Evans and Till, 1979, sensu Lindquist et al*.* 2009.

Superfamily Dermanyssoidea Kolenati, 1859, sensu Beaulieu et al*.* 2011.

Subfamily Varroinae **subfam. nov.**

Type genus *Varroa* Oudemans, 1904.

Genera included. *Varroa* Oudemans, 1904; *Euvarroa* Delfinado and Baker, 1974.

*Diagnosis.* strongly flattened, hypertrichous idiosoma, with extra setae present on the genital shield, peritremes looped and shifted to a position lateral to the stigma, subcapitulum with three pairs hypostomal setae arranged in a line (internal hypostomal setae (*h3*) absent), fixed digit of chelicera reduced and legs conspicuously shorter than idiosoma.

## Methods

### Mitochondrial genome phylogeny

The major goal of sampling strategy was to determine the phylogenetic position of *Varroa* mites by using a wide range of Dermanyssoidea mites. Therefore, 47 sequences and 25 novel sequences were obtained including three *Varroa* mites from two genera: *Varroa destructor*, *V. jacobsoni, and Euvarroa sinhai*. In total, 11 species of Dermanyssodea and 13 outgroup species (Celaenopsoidea, Eviphidoidea, Parasitoidea, Phytoseioidea, and Rhodacaroidea) were used (Supplementary Table 1). For the novel sequences, DNA libraries were prepared using the Nextera DNA Flex Library Preparation Kit (Illumina Inc., Cambridge, UK) and sequenced on an Illumina Novaseq 6000S4 (Illumina Inc.). The raw reads from the whole-genome sequences were assembled into contigs using Velvet 1.2.10. Among the contigs, those with high-GC ratio contents were screened, and ambiguous contigs were manually generated. Additionally, 77 data belonging to parasitiformes served as mitogenome reference sequences and were downloaded from NCBI Genbank (https://www.ncbi.nlm.nih.gov/genbank/). The public sequences were aligned using MAFFT 7.149 and extracted separately through Geneious Prime 2023.02.02 (https://www.geneious.com). All 13 protein-coding genes (atp6, atp8, COX1, COX2, COX3, CYTB, NAD1, NAD2, NAD3, NAD4, NAD4L, NAD5, NAD6) annotated with the MITOS Web Server^48^ and used. For three important taxa that lack full-mitogenome data, only COI sequences were used: *Euvarroa sinhai*, *Laelaps agilis*, and *L. clethrionomydis*. These data were partitioned using PARTITION-FINDER2 software, and the best-fit partition schemes were applied to Maximum Likelihood (ML) and Bayesian Inference (BI) analyses. Prior to obtaining the final results, species with lower Taxonomic Instability Index (Tii) > 0.4 or Leaf Stability Index (Lsi) < 0.9 were removed using the RoughNaRok web server^49^.

Phylogenetic analyses were conducted using both ML and BI methods. The ML analysis was carried out using IQ-TREE^50^ with 1,000 replicates of ultrafast bootstrap approximation (UFB) with the best partition scheme and the best-fit substitution models found by the PARTITION-FINDER2^51^. Bootstrap values were designated as moderate (≥ 85) and strong (≥ 95). The Bayesian Inference analysis was conducted using MRBAYES v.3.2.7^52^, the analysis was performed using 20 million Markov Chain Monte Carlo (MCMC) generations, and trees were sampled every 1000 generations. A burnin of 25% of the sampled trees was applied to ensure adequate mixing of the MCMC chain. Both analyses results were visualized using FIGTREE v.1.4.4.^53^.

### Multi-locus phylogeny

Two species of *Varroa* mites were included in multi-locus phylogeny: *V. destructor and V. jacobsoni*. A total of 108 species of Dermanyssoidea were used for the ingroup, with 94 species belonging to the family Laelapidae. The outgroup consisted of two species of Ologamasidae (rhodacaroidea) and three species of Eviphididae (eviphidoidea), which were selected based on previous studies^20–22^. Public sequences of 73 species were obtained from Genbank and those of 128 sequences from 40 species were newly added to the database (Supplementary Table 2). Newly sequenced mites were collected using Berlese-Tullgren funnel traps (60w/48 h) from various regions in Korea. Voucher specimens for identification were made after the experiment by Poly Vinyl Alcohol (PVA) mounting fluid^54^ and DNAs were stored in − 20 °C conditions. All the DNA vouchers and voucher specimens were deposited in the Insect Biosystematics Laboratory at Seoul National University.

Total genomic DNAs were extracted from Dermanyssoidea mites using DNeasy Blood & Tissue kit (Quiagen, Inc.) following the manufacturer's protocol. Four genes commonly used in Araneae phylogenetics were selected, including one nuclear protein-coding gene (H3) and three ribosomal genes (28S rDNA, 18S rDNA, ITS) which were used in previous studies^22,23,55–57^. The sequences of primers and PCR conditions are provided (Supplementary Table 3). PCR products were purified and sequenced at Bionics Inc. The raw sequence data were assembled and checked using SEQMAN PRO v.7.1.0 (DNASTAR, Madison, WI, USA). The sequences were aligned using MAFFT version 7^58^ and adjusted by MUSCLE in MEGA v.7.0.26. The aligned sequences were combined using SEQUENCEMATRIX v.1.7.8^59^. ML and BI analysis were performed the same as in Mitogenome phylogenetics.

### Ancestral character states reconstruction of host

The phoretic behavior was designated into seven states: free-living and phoretic to beetle, bee, ant, vertebrates, roaches, and diplopod. Mites that were observed on the soil surface, within animal nests, or were never found attached to the bodies of other organisms under any circumstances were categorized as 'free-living.' This classification aligns with the common lifestyle observed in many acarine species, as suggested by Krantz and Walter^60^. In contrast to the free-living category, mites with previous records of phoretic behavior or detachment from other animals' bodies. The 'ant' state includes mites detaching from ant bodies directly, within the nest, or even close to nest entrances. In total, our dataset includes 110 host designation records, excluding those marked with the '?' symbol (Supplementary Table 4).

The hosts of Laelapidae were coded as seven discrete states by their phoretic preference: (A) free-living, (B) beetle, (C) bee, (D) ant, (E) vertebrates, (F) roaches, (G) diplopod. The probability of the ancestral state of each node was calculated by Bayestraits V.4.0.0^61^ using reversible jump Markov Chain Monte Carlo (RJ-MCMC). An exponential distribution was implemented, seeding from a uniform prior in an interval of 0–100. 50 million iterations were done, sampling every 1000th iteration. The first million iterations were discarded as burn-in. Acceptance rates were automatically adjusted and achieved in the preferred range of nearly 35%. Parsimony-based ancestral character state reconstruction was conducted using the trace character over trees option in Mesquite 3.81^62^ on a single Bayesian Inference (BI) based tree.

### Supplementary Information

Supplementary Figures.

Supplementary Tables.

## Data Availability

The authors declare that all other data supporting the findings of this study are available within the supplementary information files.
