# Supplementary material for: Molecular phylogeny reveals Varroa mites are not a separate family but a subfamily of Laelapidae
Source: Sci Rep. 2024 Jun 18;14:13994. doi: 10.1038/s41598-024-63991-z (PMC11183080; doi:10.1038/s41598-024-63991-z)

Molecular phylogeny reveals *Varroa* mites are not a separate family but a subfamily of Laelapidae

Jaeseok Oh[a, +], Seunghyun Lee[a,b,c, +], Woochan Kwon[d], Omid Joharchi[e,f,g], Sora Kim[h,i], Seunghwan Lee[a, b,*]

[a] Insect Biosystematics Laboratory, Department of Agricultural Biotechnology, Seoul National University, 1, Gwanak-ro, Gwanak-gu, Seoul, Republic of Korea

[b] Research Institute of Agriculture and Life Sciences, Seoul National University, Seoul, Republic of Korea

[c] Department of Life Sciences, Natural History Museum, London, United Kingdom

[d] Division of Environmental Science and Ecological Engineering, Korea University, Seoul, Korea

[e] All-Russian Institute of Plant Protection, St. Petersburg, Russia.

[f] Agriculture Science and Technology Institute, Andong National University, Andong, Republic of Korea

[g] Johann Friedrich Blumenbach Institute of Zoology and Anthropology Animal Ecology, Georg-August-Universität-Göttingen, Göttingen, Germany

[h] Lab. of Insect phylogenetics and evolution, Department of Plant Protection & Quarantine, Jeonbuk National University, Jeonju, 54896, Republic of Korea

[i] Department of Agricultural Convergence Technology, Jeonbuk National University, Jeonju, 54896, Republic of Korea

+ Equal contribution

*Corresponding author: E-mail, seung@snu.ac.kr; Tel, +82-2-880-4703

Supplementary figure 1. Line drawing plates of *Varroa destructor*. The Genus *Varroa* was elevated to the family level based on unique morphological characteristics such as hairy dorsal and ventral shield, looped stigma, modified leg chaetotaxy, and reduced cheliceral digit.

(A)

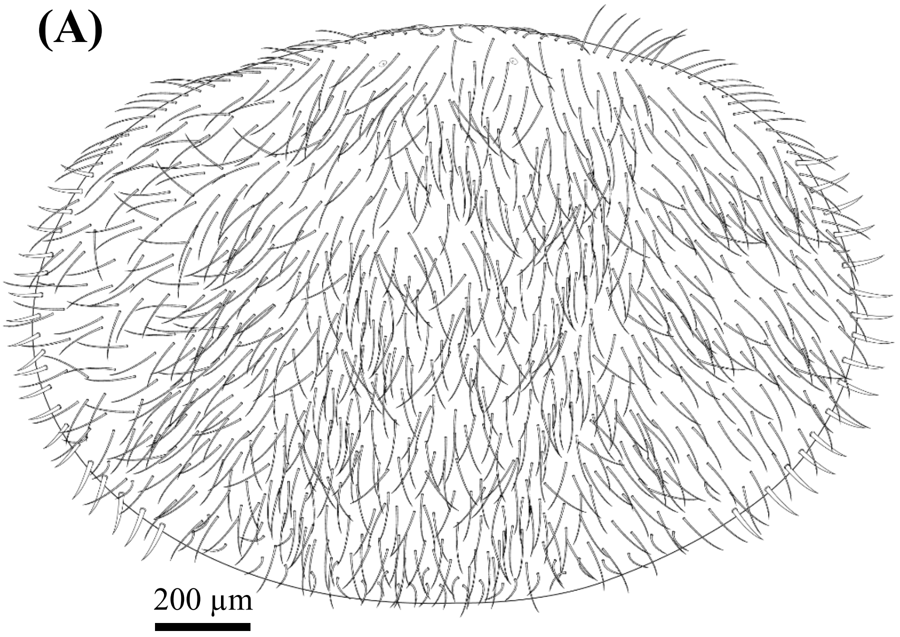

(A) dorsal view of female

(B)

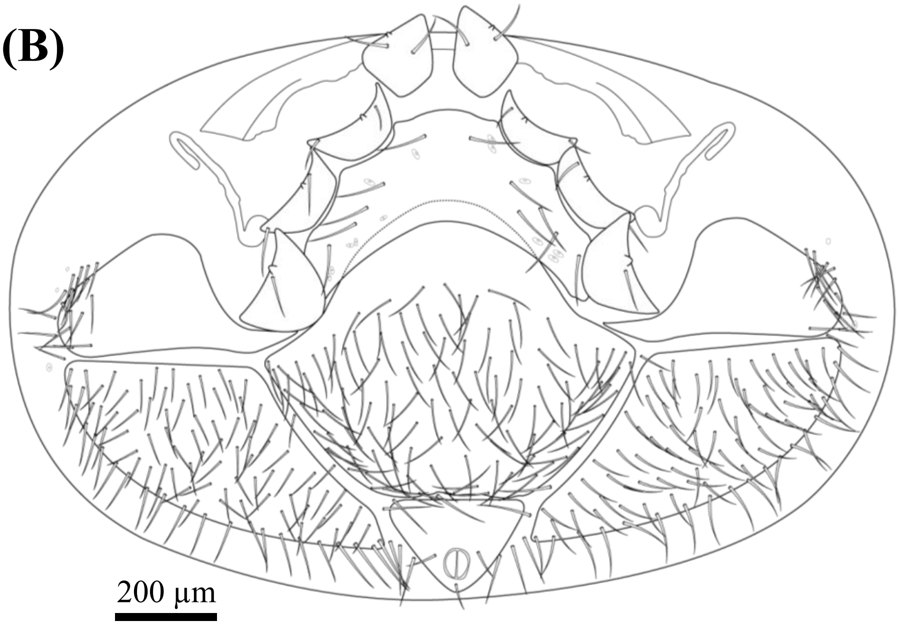

(B) ventral view of female

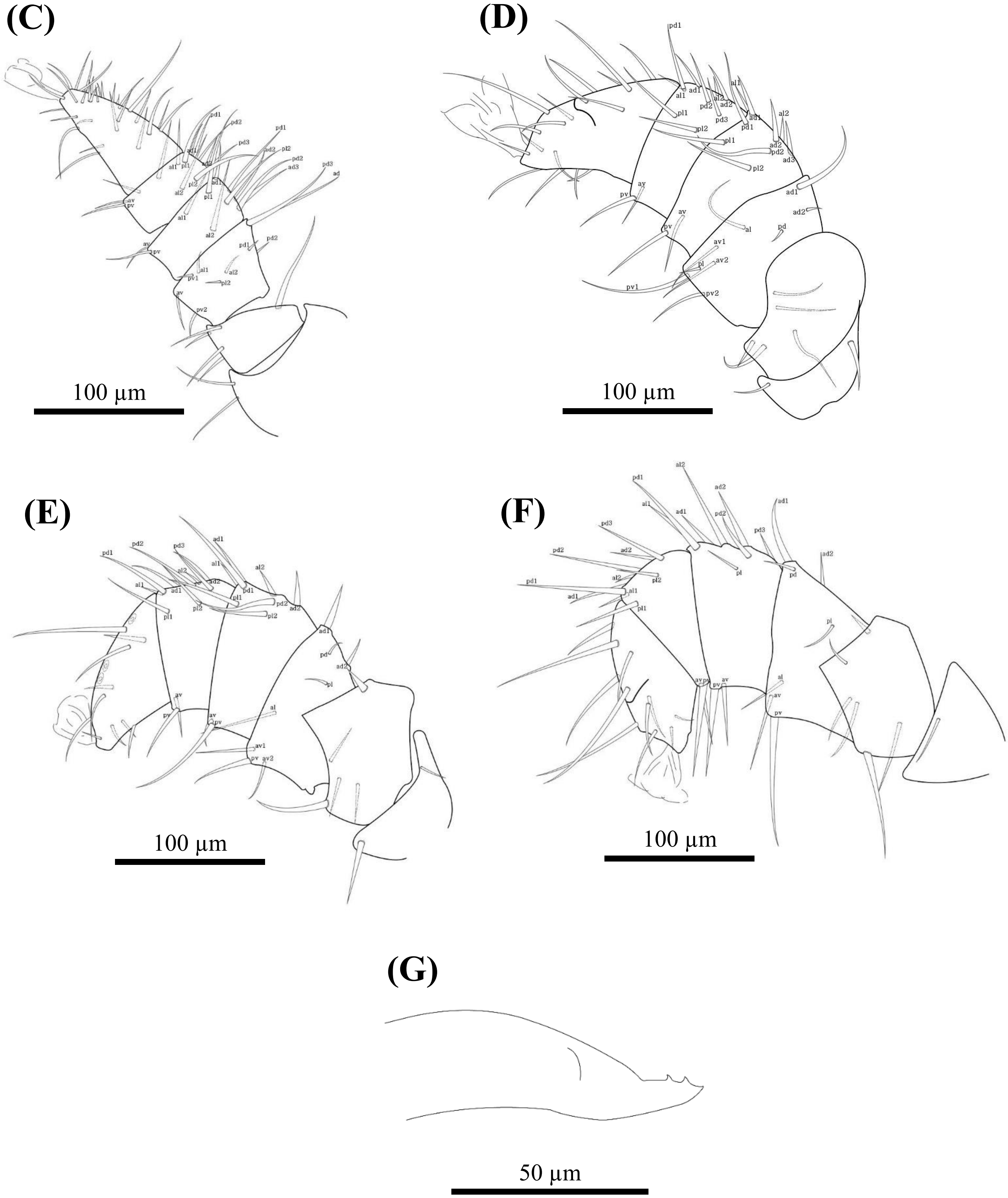

(C–G) (C) leg I; (D) leg II; (E) leg III; (F) leg IV; (G) cheliceral digit of female

Supplementary figure 2. Bayesian Inference phylogenetic tree based on Mitogenome sequences. The tree was reconstructed in MRBAYES v.3.2.7[52] with 13 mitochondrial protein-coding genes. The analysis ran for 20 million Markov chain Monte Carlo generations, with trees sampled every 100 generations. Each value represents the posterior probability of the analysis. Taxa used as an out group: from *Blattisocius tarsalis* to *Parasitus wangdunqingi.*

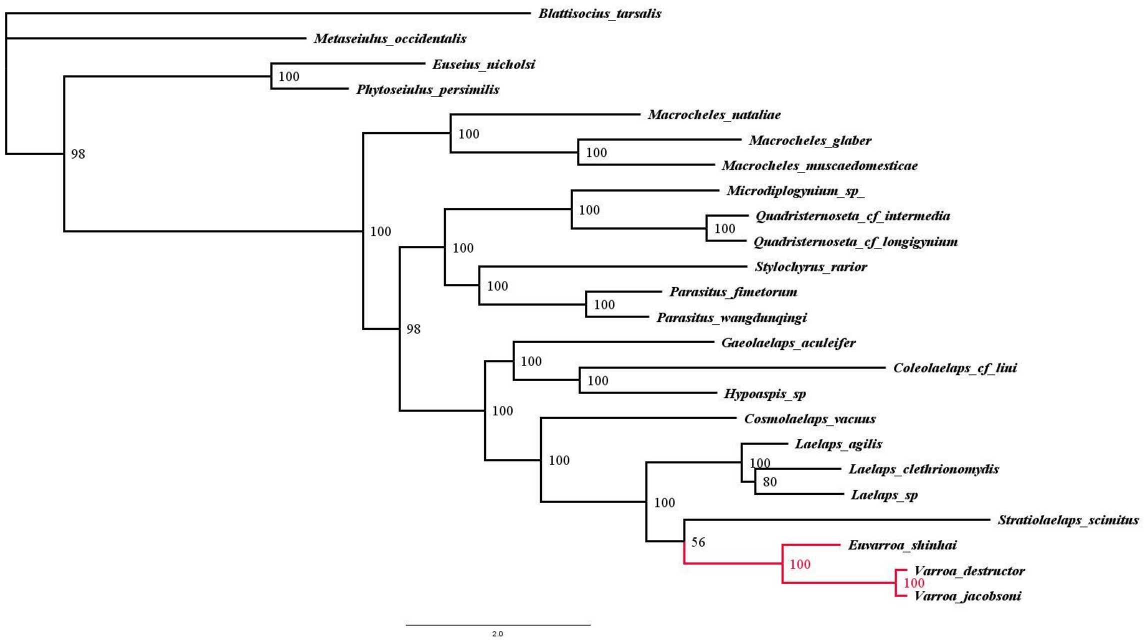

Supplementary figure 3. Maximum Likelihood phylogenetic tree based on Mitogenome sequences. The tree was reconstructed in IQ-TREE[50] with 13 mitochondrial protein-coding genes, employing the GTR+I+G, GTR+G model, along with 1,000 replicates of ultrafast bootstrap approximation. Taxa used as an out group: from *Blattisocius tarsalis* to *Parasitus wangdunqingi.*

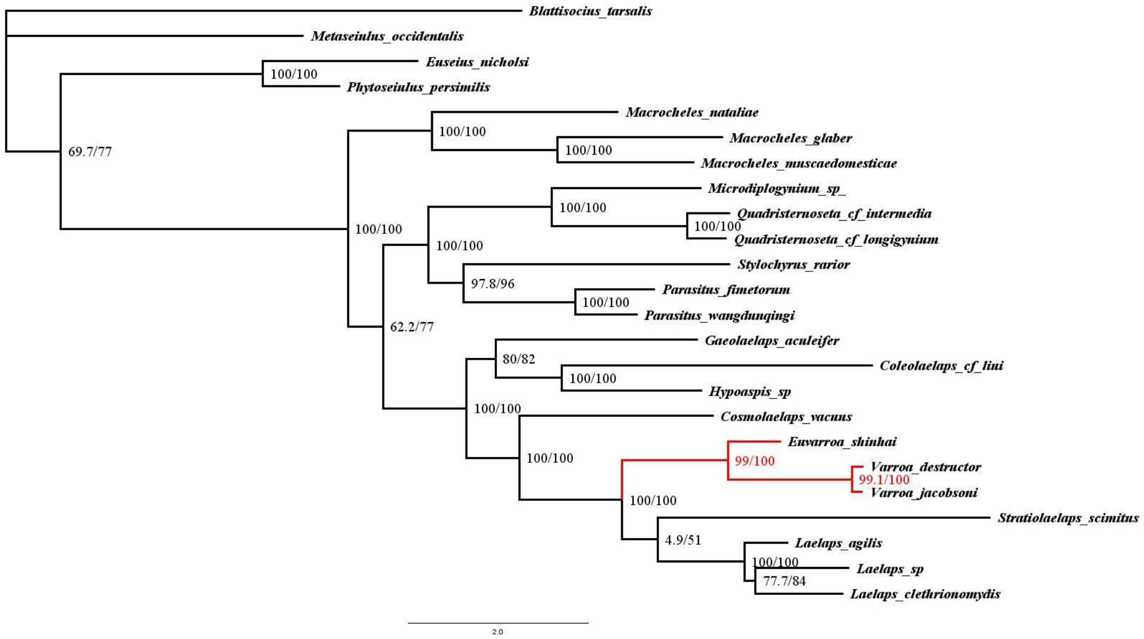

Supplementary figure 4. Bayesian Inference phylogenetic tree based on Sanger sequences. The tree was reconstructed in MRBAYES v.3.2.7[52] with four nuclear loci. The analysis ran 20 million Markov chain Monte Carlo generations, with trees sampled every 100 generations. Each value represents the posterior probability of the analysis. Taxa used as an out group: from *Alliphis necrophilus* to *Dermanyssus quintus;* from *Radfordiella oudemansi* to *Ornithonyssus wernecki.*

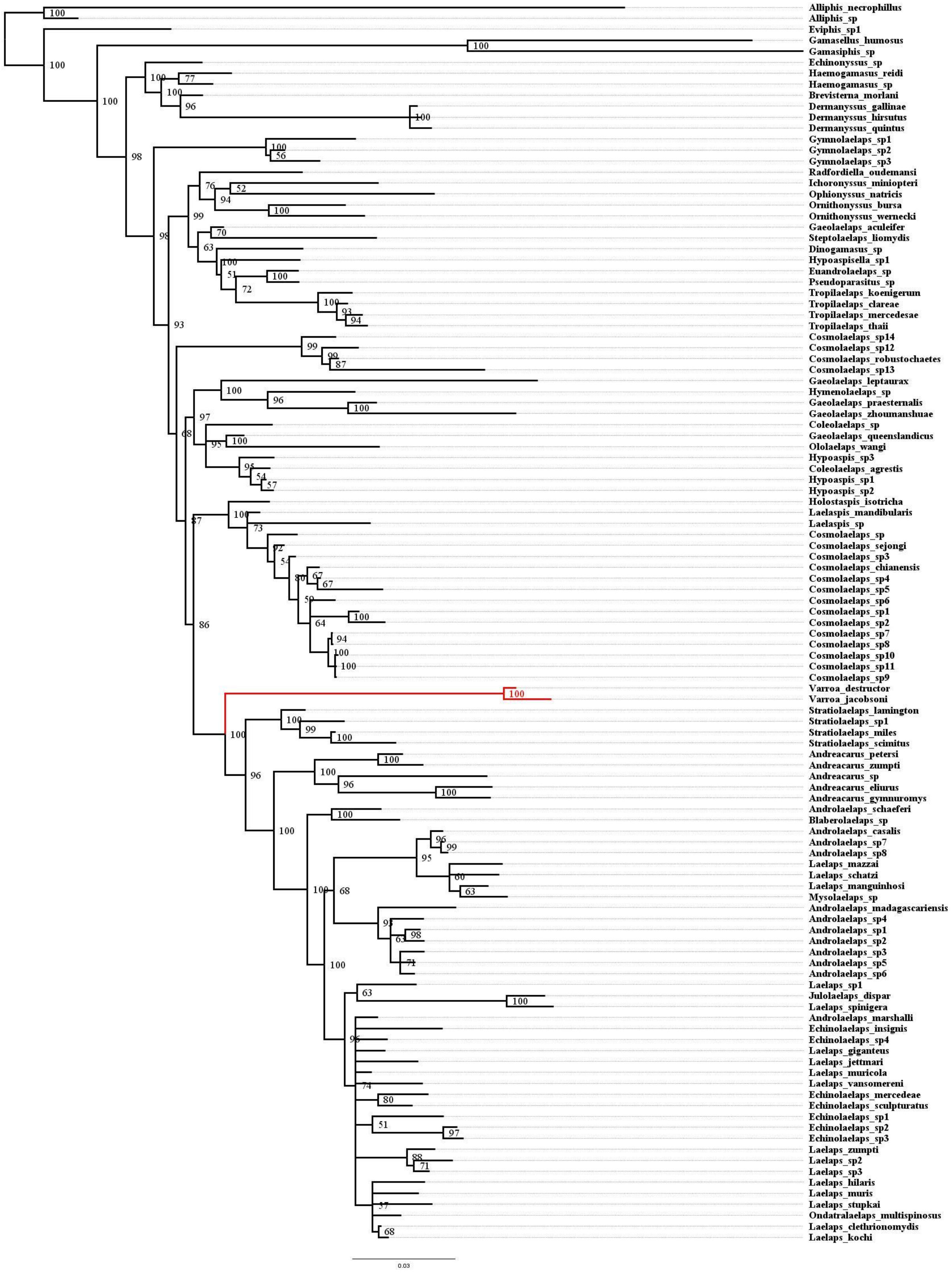

Supplementary figure 5. Maximum Likelihood phylogenetic tree based on Sanger sequences. The tree was reconstructed in IQ-TREE[50] with four nuclear loci, employing the GTR, GTR+I+G, and GTR+G models, along with 1,000 replicates of ultrafast bootstrap approximation. Taxa used as an out group: from *Alliphis necrophilus* to *Dermanyssus quintus;* from *Radfordiella oudemansi* to *Ornithonyssus wernecki.*

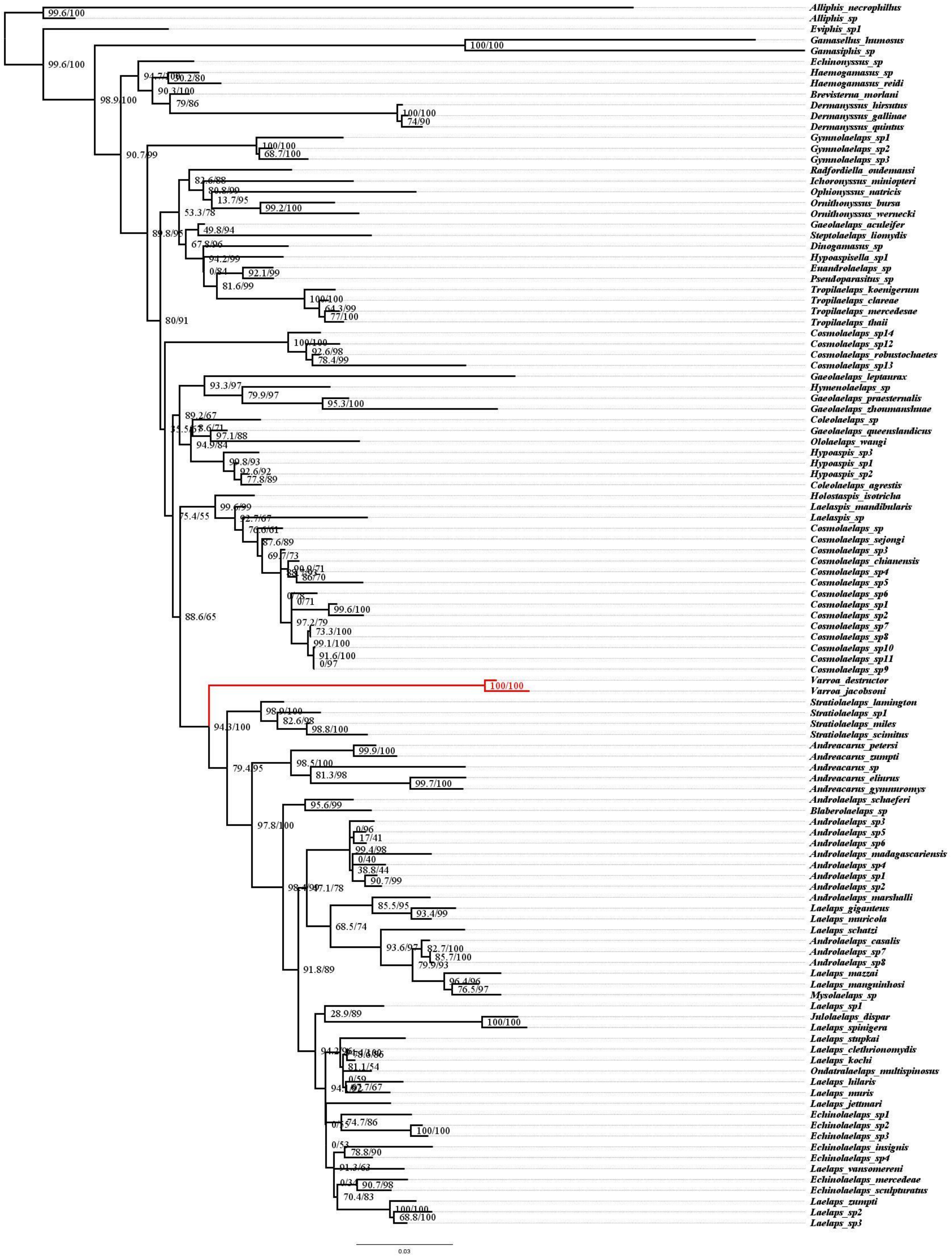

Supplement: Supplementary file 1 — Supplementary Figures. [file 41598_2024_63991_MOESM1_ESM.pdf]
