# Supplementary material for: Molecular phylogeny reveals Varroa mites are not a separate family but a subfamily of Laelapidae
Source: Sci Rep. 2024 Jun 18;14:13994. doi: 10.1038/s41598-024-63991-z (PMC11183080; doi:10.1038/s41598-024-63991-z)
Supplement: Supplementary file 2 — Supplementary Tables. [file 41598_2024_63991_MOESM2_ESM.pdf]

Molecular phylogeny reveals *Varroa* mites are not a separate family but a subfamily of Laelapidae

Jaeseok Oh[a, +], Seunghyun Lee[a,b,c, +], Woochan Kwon[d], Omid Joharchi[e,f,g], Sora Kim[h,i], Seunghwan Lee[a, b,*]

[a] Insect Biosystematics Laboratory, Department of Agricultural Biotechnology, Seoul National University, 1, Gwanak-ro, Gwanak-gu, Seoul, Republic of Korea

[b] Research Institute of Agriculture and Life Sciences, Seoul National University, Seoul, Republic of Korea

[c] Department of Life Sciences, Natural History Museum, London, United Kingdom

[d] Division of Environmental Science and Ecological Engineering, Korea University, Seoul, Korea

[e] All-Russian Institute of Plant Protection, St. Petersburg, Russia.

[f] Agriculture Science and Technology Institute, Andong National University, Andong, Republic of Korea

[g] Johann Friedrich Blumenbach Institute of Zoology and Anthropology Animal Ecology, Georg-August-Universität-Göttingen, Göttingen, Germany

[h] Lab. of Insect phylogenetics and evolution, Department of Plant Protection & Quarantine, Jeonbuk National University, Jeonju, 54896, Republic of Korea

[i] Department of Agricultural Convergence Technology, Jeonbuk National University, Jeonju, 54896, Republic of Korea

+ Equal contribution

*Corresponding author: E-mail, seung@snu.ac.kr; Tel, +82-2-880-4703

Supplementary table 1. List of the species and Genbank accession numbers included for this mitogenome study

<table>
<tr><th>Superfamily</th><th>Family</th><th>Species</th><th>ATP6</th><th>ATP8</th><th>COX1</th><th>COX2</th><th>COX3</th><th>CYTB</th><th>NAD1</th><th>NAD2</th><th>NAD3</th><th>NAD4</th><th>NAD4L</th><th>NAD5</th><th>NAD6</th></tr>
<tr><td>Celaenopsoidea</td><td>Diplogyniidae</td><td>Microdiplogynium sp.</td><td>MK270523</td><td>N.A.</td><td colspan="11">MK270523</td></tr>
</table>

<table>
<tr><td rowspan="2"></td><td rowspan="2"></td><td>Quadristernoseta cf. intermedia</td><td colspan="13">MK270521</td></tr>
<tr><td>Quadristernoseta cf. longigynium</td><td colspan="13">KM270522</td></tr>
<tr><td rowspan="12">Dermanyssoidea</td><td rowspan="9">Laelapidae</td><td>Coleolaelaps cf. liui</td><td>MK270524</td><td>N.A.</td><td colspan="11">MK270524</td></tr>
<tr><td>Cosmolaelaps vacuus</td><td colspan="5">MN622245.1</td><td>N.A.</td><td colspan="7">MN622245.1</td></tr>
<tr><td>Gaeolaelaps aculeifer</td><td colspan="5">MN622222.1</td><td>N.A.</td><td colspan="7">MN622222.1</td></tr>
<tr><td>Hypoaspis sp</td><td>OR399764</td><td>OR399766</td><td>OR506907</td><td>OR399768</td><td>OR399770</td><td>OR399772</td><td>OR399774</td><td>OR421506</td><td>OR421508</td><td>N.A.</td><td>OR421511</td><td>OR421513</td><td>OR421515</td></tr>
<tr><td>Laelaps agilis</td><td colspan="2">N.A.</td><td>OM754646.1</td><td>N.A.</td><td>OM754646.1</td><td colspan="8">N.A.</td></tr>
<tr><td>Laelaps clethrionomydis</td><td colspan="2">N.A.</td><td>OM754650.1</td><td colspan="10">N.A.</td></tr>
<tr><td>Laelaps sp</td><td>OR399765</td><td>OR399767</td><td>OR506908</td><td>OR399769</td><td>OR399771</td><td>OR399773</td><td>OR399775</td><td>OR421507</td><td>OR421509</td><td>OR421510</td><td>OR421512</td><td>OR421514</td><td>OR421516</td></tr>
<tr><td>Stratiolaelaps scimitus</td><td colspan="5">MN781133</td><td>N.A.</td><td colspan="2">MN781133</td><td>N.A.</td><td colspan="2">MN781133</td><td colspan="2">N.A.</td></tr>
<tr><td>Euvarroa sinhai</td><td colspan="2">N.A.</td><td>MW585685.1</td><td colspan="10">N.A.</td></tr>
<tr><td rowspan="3">Varroidae</td><td>Varroa destructor</td><td colspan="13">NC004454</td></tr>
<tr><td>Varroa jacobsoni</td><td>N.A.</td><td colspan="4">SRX2940461 (SRA assemble)</td><td>N.A.</td><td colspan="7">SRX2940461 (SRA assemble)</td></tr>
<tr><td rowspan="3">Eviphidoidea</td><td rowspan="3">Macrochelidae</td><td>Macrocheles glaber</td><td colspan="7">MK270525</td><td>N.A.</td><td colspan="4">MK270525</td><td>N.A.</td></tr>
<tr><td>Macrocheles muscaedomesticae</td><td colspan="13">MK270526</td></tr>
<tr><td>Macrocheles nataliae</td><td colspan="2">MK270527</td><td>N.A.</td><td colspan="7">MK270527</td><td>N.A.</td><td colspan="2">MK270527</td></tr>
<tr><td rowspan="2">Parasitoidea</td><td rowspan="2">Parasitidae</td><td>Parasitus fimetorum</td><td colspan="13">OK572962</td></tr>
<tr><td>Parasitus wangdunqingi</td><td colspan="12">MK270528</td><td>N.A.</td></tr>
<tr><td rowspan="4">Phytoseioidea</td><td>Blattisociidae</td><td>Blattisocius tarsalis</td><td colspan="13">MK270529</td></tr>
<tr><td rowspan="3">Phytoseiidae</td><td>Euseius nicholsi</td><td colspan="7">KM999989</td><td>N.A.</td><td colspan="5">KM999989</td></tr>
<tr><td>Metaseiulus occidentalis</td><td colspan="2">EF221760</td><td>N.A.</td><td colspan="5">EF221760</td><td>N.A.</td><td colspan="3">EF221760</td><td>N.A.</td></tr>
<tr><td>Phytoseiulus persimilis</td><td colspan="13">GQ222414</td></tr>
<tr><td>Rhodacaroidea</td><td>Ologamasidae</td><td>Stylochyrus rarior</td><td colspan="10">GQ927176</td><td>N.A.</td><td colspan="2">GQ927176</td></tr>
</table>

*Red marked data: novel sequences

Supplementary table 2. List of the species and Genbank accession number included in multi-locus phylogeny.

Family	Subfamily	Species	GeneBank accession number				Specimen Reference
			18S	28S	ITS	H3	
Dermanyssidae		*Dermanyssus gallinae*	OR298172	OR298135	OR348752	OR343154	P
		Dermanyssus hirsutus	N.A.	GU440633	N.A.	N.A.	N
		Dermanyssus quintus	N.A.	FJ911769	N.A.	N.A.	N
Eviphididae		*Alliphis necrophillus*	OR298157	OR298117	OR348749	OR343140	P
		Alliphis sp.	FJ911818	FJ911753	N.A.	N.A.	N
		Eviphis sp.1	N.A.	FJ911754	N.A.	N.A.	N
Haemogamasidae		*Brevisterna morlani*	FJ911838	FJ911773	N.A.	N.A.	N
		Haemogamasus reidi	N.A.	GU440583	N.A.	N.A.	N
		Haemogamasus sp.	FJ911837	FJ911772	N.A.	N.A.	N
Hirstionyssidae		*Echinonyssus* sp.	FJ911840	FJ911775	N.A.	N.A.	N
Laelapidae	Iphiopsidinae	*Julolaelaps dispar*	N.A.	GU440603	N.A.	N.A.	N
	Laelapinae	*Andreacarus eliurus*	N.A.	GU440617	N.A.	N.A.	N
	Laelapinae	*Andreacarus gymnuromys*	N.A.	GU440618	N.A.	N.A.	N
	Laelapinae	*Andreacarus petersi*	FJ911847	FJ911782	N.A.	N.A.	N
	Laelapinae	*Andreacarus* sp.	N.A.	GU440598	N.A.	N.A.	N
	Laelapinae	*Andreacarus zumpti*	N.A.	GU440629	N.A.	N.A.	N
	Hypoaspidinae	*Androlaelaps casalis*	N.A.	GU440582	AM903317.1	N.A.	N
	Hypoaspidinae	*Androlaelaps madagascariensis*	FJ911849	FJ911784	N.A.	N.A.	N
	Hypoaspidinae	*Androlaelaps marshalli*	N.A.	N.A.	MF419349.1	N.A.	N
	Hypoaspidinae	*Androlaelaps schaeferi*	FJ911844	FJ911779	N.A.	N.A.	N
	Hypoaspidinae	*Androlaelaps* sp.1	N.A.	GU440624	N.A.	N.A.	N
	Hypoaspidinae	*Androlaelaps* sp.2	N.A.	GU440625	N.A.	N.A.	N
	Hypoaspidinae	*Androlaelaps* sp.3	N.A.	GU440597	N.A.	N.A.	N

Hypoaspidinae	*Androlaelaps* sp.4	N.A.	GU440627	N.A.	N.A.	N
Hypoaspidinae	*Androlaelaps* sp.5	N.A.	GU440622	N.A.	N.A.	N
Hypoaspidinae	*Androlaelaps* sp.6	N.A.	GU440621	N.A.	N.A.	N
Hypoaspidinae	*Androlaelaps* sp.7	N.A.	GU440592	N.A.	N.A.	N
Hypoaspidinae	*Androlaelaps* sp.8	N.A.	GU440601	N.A.	N.A.	N
Hypoaspidinae	*Blaberolaelaps* sp.	FJ911850	FJ911785	N.A.	N.A.	N
Hypoaspidinae	*Coleolaelaps agrestis*	N.A.	N.A.	DQ9863811	N.A.	N
Hypoaspidinae	*Coleolaelaps* sp.	N.A.	GU440607	N.A.	N.A.	N
Hypoaspidinae	*Cosmolaelaps chianensis*	OR298158	OR298118	OR348750	OR343141	P
Hypoaspidinae	*Cosmolaelaps robustochaetes*	OR298159	OR298119	N.A.	OR343142	P
Hypoaspidinae	*Cosmolaelaps sejongi*	OR298160	OR298120	OR348751	N.A.	P
Hypoaspidinae	*Cosmolaelaps* sp.	N.A.	GU440606	N.A.	N.A.	N
Hypoaspidinae	*Cosmolaelaps* sp.1	OR298161	OR298121	N.A.	OR343143	P
Hypoaspidinae	*Cosmolaelaps* sp.10	OR298162	OR298122	N.A.	OR343144	P
Hypoaspidinae	*Cosmolaelaps* sp.11	OR298163	OR298123	N.A.	OR343145	P
Hypoaspidinae	*Cosmolaelaps* sp.12	OR298164	OR298124	N.A.	OR343146	P
Hypoaspidinae	*Cosmolaelaps* sp.13	N.A.	OR298125	N.A.	OR343147	P
Hypoaspidinae	*Cosmolaelaps* sp.14	OR298165	OR298126	N.A.	OR343148	P
Hypoaspidinae	*Cosmolaelaps* sp.2	N.A.	OR298127	N.A.	N.A.	P
Hypoaspidinae	*Cosmolaelaps* sp.3	N.A.	OR298128	N.A.	N.A.	P
Hypoaspidinae	*Cosmolaelaps* sp.4	OR298166	OR298129	N.A.	OR343149	P
Hypoaspidinae	*Cosmolaelaps* sp.5	OR298167	OR298130	N.A.	OR343150	P
Hypoaspidinae	*Cosmolaelaps* sp.6	OR298168	OR298131	N.A.	OR343151	P
Hypoaspidinae	*Cosmolaelaps* sp.7	OR298169	OR298132	N.A.	OR343152	P
Hypoaspidinae	*Cosmolaelaps* sp.8	OR298170	OR298133	N.A.	N.A.	P
Hypoaspidinae	*Cosmolaelaps* sp.9	OR298171	OR298134	N.A.	OR343153	P
Melittiphidinae	*Dinogamasus* sp.	FJ911845	FJ911780	N.A.	N.A.	N

Laelapinae	*Echinolaelaps insignis*	N.A.	GU440588	N.A.	N.A.	N
Laelapinae	*Echinolaelaps mercedeae*	N.A.	GU440593	N.A.	N.A.	N
Laelapinae	*Echinolaelaps sculpturatus*	N.A.	GU440587	N.A.	N.A.	N
Laelapinae	*Echinolaelaps* sp.1	N.A.	GU440610	N.A.	N.A.	N
Laelapinae	*Echinolaelaps* sp.2	N.A.	GU440599	N.A.	N.A.	N
Laelapinae	*Echinolaelaps* sp.3	N.A.	GU440600	N.A.	N.A.	N
Laelapinae	*Echinolaelaps* sp.4	N.A.	GU440611	N.A.	N.A.	N
Hypoaspidinae	*Euandrolaelaps* sp.	FJ911846	FJ911781	N.A.	N.A.	N
Hypoaspidinae	*Gaeolaelaps aculeifer*	OR298173	OR298136	N.A.	N.A.	P
Hypoaspidinae	*Gaeolaelaps praesternalis*	OR298174	OR298137	OR348753	OR343155	P
Hypoaspidinae	*Gaeolaelaps queenslandicus*	OR298175	OR298138	OR348754	OR343156	P
Hypoaspidinae	*Gaeolaelaps leptaurax*	OR298176	OR298139	OR348755	OR343157	P
Hypoaspidinae	*Gaeolaelaps zhoumanshuae*	OR298177	OR298140	N.A.	N.A.	P
Melittiphidinae	*Gymnolaelaps* sp.1	OR298180	OR298143	OR348757	OR343160	P
Melittiphidinae	*Gymnolaelaps* sp.2	OR298181	OR298144	OR348758	OR343161	P
Melittiphidinae	*Gymnolaelaps* sp.3	OR298182	OR298145	N.A.	OR343162	P
Melittiphidinae	*Holostaspis isotricha*	FJ911851	FJ911786	N.A.	N.A.	N
Laelapinae	*Hymenolaelaps* sp	N.A.	GU440631	N.A.	N.A.	N
Hypoaspidinae	*Hypoaspis* sp.1	OR298183	OR298146	OR348759	OR343163	P
Hypoaspidinae	*Hypoaspis* sp.2	N.A.	OR298147	OR348760	OR343164	P
Hypoaspidinae	*Hypoaspis* sp.3	OR298184	OR298148	N.A.	OR343165	P
Laelapinae	*Laelaps clethrionomydis*	N.A.	GU440636	N.A.	N.A.	N
Laelapinae	*Laelaps giganteus*	N.A.	N.A.	MF419313.1	N.A.	N
Laelapinae	*Laelaps hilaris*	N.A.	GU440637	N.A.	N.A.	N
Laelapinae	*Laelaps jettmari*	N.A.	GU440635	N.A.	N.A.	N
Laelapinae	*Laelaps kochi*	N.A.	GU440626	N.A.	N.A.	N
Laelapinae	*Laelaps manguinhosi*	N.A.	GU440591	N.A.	N.A.	N

Laelapinae	*Laelaps mazzai*	N.A.	GU440590	N.A.	N.A.	N
Laelapinae	*Laelaps muricola*	N.A.	N.A.	MF419348.1	N.A.	N
Laelapinae	*Laelaps muris*	N.A.	GU440638	N.A.	N.A.	N
Laelapinae	*Laelaps schatzi*	N.A.	N.A.	MK725870.1	N.A.	N
Laelapinae	*Laelaps* sp.1	N.A.	GU440628	N.A.	N.A.	N
Laelapinae	*Laelaps* sp.2	N.A.	GU440602	N.A.	N.A.	N
Laelapinae	*Laelaps* sp.3	N.A.	GU440608	N.A.	N.A.	N
Laelapinae	*Laelaps spinigera*	N.A.	GU440613	N.A.	N.A.	N
Laelapinae	*Laelaps stupkai*	N.A.	GU440596	N.A.	N.A.	N
Laelapinae	*Laelaps vansomereni*	N.A.	GU440619	N.A.	N.A.	N
Laelapinae	*Laelaps zumpti*	N.A.	GU440623	N.A.	N.A.	N
Melittiphidinae	*Laelaspis mandibularis*	OR298185	OR298149	OR348761	OR343166	P
Melittiphidinae	*Laelaspis* sp.	N.A.	FJ911783	N.A.	N.A.	N
Hypoaspidinae	*Hypoaspisella* sp.1	OR298186	OR298150	N.A.	OR343167	P
Laelapinae	*Mysolaelaps* sp.	N.A.	GU440632	N.A.	N.A.	N
Hypoaspidinae	*Ololaelaps wangi*	OR298187	OR298151	OR348762	OR343168	P
Laelapinae	*Ondatralaelaps multispinosus*	FJ911843	FJ911778	N.A.	N.A.	N
Hypoaspidinae	*Pseudoparasitus* sp.	N.A.	GU440584	N.A.	N.A.	N
Laelapinae	*Steptolaelaps liomydis*	N.A.	GU440859	N.A.	N.A.	N
Hypoaspidinae	*Stratiolaelaps lamington*	N.A.	GU440604	N.A.	N.A.	N
Hypoaspidinae	*Stratiolaelaps miles*	OR298188	OR298152	OR348763	OR343169	P
Hypoaspidinae	*Stratiolaelaps scimitus*	OR298189	OR298153	OR348764	OR343170	P
Hypoaspidinae	*Stratiolaelaps* sp.1	OR298190	OR298154	OR348765	OR343171	P
Melittiphidinae	*Tropilaelaps clareae*	N.A.	N.A.	EF025474.1	N.A.	N
Melittiphidinae	*Tropilaelaps koenigerum*	N.A.	N.A.	EF025475.1	N.A.	N
Melittiphidinae	*Tropilaelaps mercedesae*	OR298191	OR298155	OR348766	OR343172	P
Melittiphidinae	*Tropilaelaps thaii*	N.A.	N.A.	EF025477.1	N.A.	N

Macronyssidae	*Ichoronyssus miniopteri*	N.A.	FJ911791	N.A.	N.A.	N
	Ophionyssus natricis	N.A.	FJ911788	N.A.	N.A.	N
	Ornithonyssus bursa	N.A.	FJ911789	N.A.	N.A.	N
	Ornithonyssus wernecki	N.A.	GU440630	N.A.	N.A.	N
	Radfordiella oudemansi	N.A.	GU440615	N.A.	N.A.	N
Ologamasidae	*Gamasellus humosus*	OR298178	OR298141	OR348756	OR343158	P
	Gamasiphis sp.	OR298179	OR298142	N.A.	OR343159	P
Varroidae	*Varroa destructor*	OR298192	OR298156	N.A.	OR343173	P
	Varroa jacobsoni	SRX2940461 (SRA assemble)				N

*P, personal collection; N, NCBI collection

Supplementary table 3. Primer sets used in this study

Genes	Direction	Primer	Sequence (5'-3')	Reference
28S[1]	Forward	43F	GCTGCGAGTGAACTGGAATCAAGCCT	Dowling & OConnor, 2010a
	Reverse	929R	AGGTCACCATTTTCGGGTC	
18S[2] (partial)	Part I (F)	NS1	GTAGTCATATGCTTGTCTC	White et al., 1990
	Part I (R)	NS2	GGCTGCTGGCACCAGACTTGC	
	Part II (F)	NS3	GCAAGTCTGGTGCCAGCAGCC	
	Part II (R)	NS4	CTTCCGTCAATTCCTTTAAG	
	Part III (F)	NS5	AACTTAAAGGAATTGACGGAAG	
	Part III (R)	NS8	TCCGCAGGTTCACCTACGGA	
ITS[3]	Forward	ITS4	TCCTCCGCTTATTGATATGC	
	Reverse	ITS5	GGAAGTAAAAGTCGTAACAAGG	
H3[4]	Forward	H3aF	ATGGCTCGTACCAAGCAGAC(ACG)GC	Colgan et al., 1998
	Reverse	H3aR	ATATCCTT(AG)GGCAT(AG)AT(AG)GTGAC	

[1]PCR conditions (28SrDNA): Initial denaturation at 94°C for 2 min, 94°C for 25 sec, 53°C for 20sec, 72°C 1min (35 cycles), Final extension at 72°C 7 min.

[2]PCR conditions Partial primers (18SrDNA I–III): Initial denaturation at 94°C for 3 min, 95°C for 30 sec, 51°C for 30sec, 72°C 45sec (35 cycles), Final extension at 72°C 10 min.

[3]PCR conditions (ITS1-5.8S-ITS2): Initial denaturation at 94°C for 5 min, 94°C for 1 min, 53.7°C for 1min, 72°C 45sec (35 cycles), Final extension at 72°C 5 min.

[4]PCR conditions (Histone3): Initial denaturation at 94°C for 5 min, 94°C for 30 sec, 50°C for 30sec, 72°C 30sec (35 cycles), Final extension at 72°C 5 min.

Supplementary table 4. List of the species and their hosts

Taxon	Host	Specific host	Reference
Alliphis necrophillus	C	*Geotrupes amoenus* / *Nicrophorus* spp.	Keum et al., 2017b / Masan and Halliday, 2010
Alliphis sp.	?	N.A.	Dowling and Oconnor, 2010
Eviphis sp.1	?	N.A.	Dowling and Oconnor, 2010
Gamasellus humosus	A	Soil and litter of a coniferous forest	Castilho et al., 2016
Gamasiphis sp.	A	Leaflitter soil	personal collection
Andreacarus eliurus	E	*Eliurus* spp. (*E. webbi*; *E. minor*; *E. tanala*)	Dowling et al., 2007
Andreacarus gymnuromys	E	*Gymnuromys roberti*	Dowling et al., 2007
Andreacarus petersi	E	*Hemimerus talpoides*; *H. hanseni*; *Cricetomys gambianus*; *C. ansorgei*; *C. kivuensis*; *C. emini*; *Arvicanthis niloticus*; *Mastomys natalensis*	Dowling et al., 2007
Andreacarus sp.	E	*Cricetomys gambianus*	Dowling and Oconnor, 2010
Andreacarus zumpti	E	*Cricetomys gambianus*	Dowling et al., 2007
Androlaelaps casalis	E	*Rattus rattus*; *Xerus inauris*; *Sciurus carolinensis*; various nest of birds and mammalians; Straw, hay, detritus etc.	Till, 1963
Androlaelaps madagascariensis	E	*Nesogale dobsoni*	Tipton, 1957
Androlaelaps marshalli	E	*Tatera afra*; *Gerbillus paeba*; *Desmodillus auricularis*; *Rattus paedulcus*; *R. natalensis*; *R. chrysophilus*; *R. namaquensis*; *Rhabdomys pumilio*; *Lemniscomys griselda*; Saccostomus campestris; *Steatomys pratensis*; *Pedetes capensis*; *Cryptomys hottentotus*; *Oryctolagus cuniculus*; *Arvicanthis niloticus*	Till, 1963
Androlaelaps schaeferi	F	*Gromphadorhina portentosa*	Till, 1969
Androlaelaps sp.1	E	Small mammals	Dowling and Oconnor, 2010
Androlaelaps sp.2	E	Small mammals	Dowling and Oconnor, 2010
Androlaelaps sp.3	E	Small mammals	Dowling and Oconnor, 2010
Androlaelaps sp.4	E	Small mammals	Dowling and Oconnor, 2010
Androlaelaps sp.5	E	Small mammals	Dowling and Oconnor, 2010
Androlaelaps sp.6	E	Small mammals	Dowling and Oconnor, 2010
Androlaelaps sp.7	A	Treehole litter	Dowling and Oconnor, 2010

Androlaelaps sp.8	D	*Formica* ant nest	Dowling and Oconnor, 2010
Blaberolaelaps sp.	F	Cockroach	Dowling and Oconnor, 2010
Brevisterna morlani	E	*Neotoma albigula algibula*	Strandmann and Allred, 1956
Coleolaelaps agrestis	B	*Polyphylla alba*; *P. boryi*; *P. fullo*; *P. oliviery*; *Sphodroxia maroccana*	Trach and Joharchi, 2018
Coleolaelaps sp.	B	Beetles	Dowling and Oconnor, 2010
Cosmolaelaps chianensis	?	*Mus pahari* (original) / moss (*C. hefeiensis* = junior synonym of *C. chianensis*)	Gu, 1990 / Xu and Liang, 1996
Cosmolaelaps robustochaetes	A	Leaves litter, bark, tree cavity	Ma and Lin, 2009
Cosmolaelaps sejongi	A	Soil under the oak tree	Keum et al., 2017a
Cosmolaelaps sp.	D	Ants	Dowling and Oconnor, 2010
Cosmolaelaps sp.1	D	Top soil near the ant nest or ant trail	personal collection
Cosmolaelaps sp.2	D	Top soil near the ant nest or ant trail	personal collection
Cosmolaelaps sp.3	D	Top soil near the ant nest or ant trail	personal collection
Cosmolaelaps sp.4	D	Top soil near the ant nest or ant trail	personal collection
Cosmolaelaps sp.5	D	Top soil near the ant nest or ant trail	personal collection
Cosmolaelaps sp.6	D	Top soil near the ant nest or ant trail	personal collection
Cosmolaelaps sp.7	D	Top soil near the ant nest or ant trail	personal collection
Cosmolaelaps sp.8	D	Top soil near the ant nest or ant trail	personal collection
Cosmolaelaps sp.9	D	Top soil near the ant nest or ant trail	personal collection
Cosmolaelaps sp.10	D	Top soil near the ant nest or ant trail	personal collection
Cosmolaelaps sp.11	D	Top soil near the ant nest or ant trail	personal collection
Cosmolaelaps sp.12	D	Nest of ants	personal collection
Cosmolaelaps sp.13	D	Nest of ants (*Aphaenogaster* sp.)	personal collection
Cosmolaelaps sp.14	D	Nest of ants	personal collection
Dermanyssus gallinae	E	*Acrocephalus arundinaceus*; *Aegolius funereus*; *Carduelis carduelis*; *C. spinus*; *Columba livia*; *Delichon urbica*; *Emberiza citrinella*; *Erithacus rubecula*; *Ficedula albicollis*; *Ficedula hypoleuca*; *Hirundo rustica*; *Jynx torquilal*; *Merops apiaster*; *Parus major*; *P. ater*; *Passer domesticus*; *P. montanus*; *Phoenicurus phoenicurus*; *Remiz pendulinus*; *Riparia riparia*; *Serinus canarius*; *Sitta europaea*; *Sturnus vulgaris*	Roy and Chauve, 2007

Dermanyssus hirsutus	E	*Colaptes cafer*	Roy and Chauve, 2007
Dermanyssus quintus	E	*Dendrocopos major*; *D. pubescens*; *Dryobates leucotes*; *D. major*; *Picoides pubescens*; *P. tridactylus*; *P. viridis*	Roy and Chauve, 2007
Dinogamasus sp.	C	Xylocopine bees	Dowling and Oconnor, 2010
Echinolaelaps insignis	E	*Rattus palawensis*; *R. exulans*; *R. panglima*; *R. rajah*	Strandtmann and Mitchell, 1963
Echinolaelaps mercedeae	E	*Rattus palawanensis*; *R. panglima*	Strandtmann and Mitchell, 1963
Echinolaelaps sculpturatus	E	*Rattus whiteheadi*	Strandtmann and Mitchell, 1963
Echinolaelaps sp.1	E	Mammal	Dowling and Oconnor, 2010
Echinolaelaps sp.2	E	Mammal	Dowling and Oconnor, 2010
Echinolaelaps sp.3	E	Mammal	Dowling and Oconnor, 2010
Echinolaelaps sp.4	E	Mammal	Dowling and Oconnor, 2010
Echinonyssus sp.	E	Mammal	Dowling and Oconnor, 2010
Euandrolaelaps sp.	A	Living in nests of arthropods and vertibrates not parasitic	Dowling and Oconnor, 2010
Gaeolaelaps aculeifer	A	Common in soil; Nest of *Riparia riparia*; nest of *Spalax ehrenbergi*; Some rodent nests	Evans and Till, 1966
Gaeolaelaps praesternalis	A	Soil; Grassland; Marshes	Evans and Till, 1966
Gaeolaelaps queenslandicus	A	Leaf debris	Womersley, 1954
Gaeolaelaps leptaurax	B	body of *Leptaurax koreanus*	Oh et al., 2023
Gaeolaelaps zhoumanshuae	A	Grassland soil; Humid land	Joharchi et al., 2019
Gymnolaelaps sp.1	A	Guano sediment in cave	personal collection
Gymnolaelaps sp.2	A	Guano sediment in cave	personal collection
Gymnolaelaps sp.3	A	Leaf litter soil	personal collection
Haemogamasus reidi	E	*Sciurus carolinensis*	Redington and Jachowski Jr, 1972
Haemogamasus sp.	E	Vertebrates	Dowling and Oconnor, 2010
Holostaspis isotricha	D	in *Formica pratensis* ant hill	Babaeian et al., 2019
Hymenolaelaps sp.	E	Caenolestid marsupials	Dowling and Oconnor, 2010
Hypoaspis sp.1	B	*Macrodorcus rectus*	personal collection
Hypoaspis sp.2	B	*Hexarthrius parryi*	personal collection
Hypoaspis sp.3	B	*Allomyrina dichtoma*	personal collection

Ichoronyssus miniopteri	E	*Miniopterus schreibersii natalensis*; *Miniopterus schreibersii blepotis*; *Coleura afra*	Radobsky, 1967
Julolaelaps dispar	G	On Julid sp.	Berlese, 1916 / Ryke, 1959
Laelaps clethrionomydis	E	*Clethrionomys* sp.; *Microtus* sp.	Vinarski and Korallo-Vinarskaya, 2016
Laelaps giganteus	E	*Rhabdomys intermedius*; *R. pumilio*; *R. bechuane*; *R. d. chkae*; *R. d. dilectus*	Engelbrecht et al., 2016
Laelaps hilaris	E	*Microtus arvalis*; *Microtus* sp.	Vinarski and Korallo-Vinarskaya, 2016
Laelaps jettmari	E	*Cricetulus griseus*; *Apodemus agrarius*; *Cricetulus* sp.	Vinarski and Korallo-Vinarskaya, 2016
Laelaps kochi	E	*Microtus* sp.; *Mus sylvaticua*; *Arvicola arvolis*; *Microtus coragaster*; *M. pennsylvanicus*; *M. chrotorrhinus*; *M. montanus*; *Lemnus arvalis*	Tipton, 1960
Laelaps manguinhosi	E	*Holochilus vulpinus*	Tipton, 1960
Laelaps mazzai	E	Wild rat	Tipton, 1960
Laelaps muricola	E	*Micaelamys namaquensis*; *M. natalensis*; *Mastomys* sp.; *M. coucha*	Matthee et al., 2018
Laelaps muris	E	*Arvicola amphibious*	Vinarski and Korallo-Vinarskaya, 2016
Laelaps schatzi	E	*Oligoryzomys flavescens*	Savchenko and Lareschi, 2019
Laelaps sp.1	E	Mammal	Dowling and Oconnor, 2010
Laelaps sp.2	E	Mammal	Dowling and Oconnor, 2010
Laelaps sp.3	E	Mammal	Dowling and Oconnor, 2010
Laelaps spinigera	E	*Rattus panglima*	Delfinado, 1960
Laelaps stupkai	E	*Synaptomys cooperi*	Linzey and Crossley, 1971
Laelaps vansomereni	E	*Dasymys* sp.; *Aethomys chrysophilus*	Tipton, 1960
Laelaps zumpti	E	*Mus triton*	Tipton, 1960
Laelaspis mandibularis	A	Riparian and grassland soil	Keum et al., 2017 / Original description (Ewing, 1909: Habitat unknown)
Laelaspis sp.	D	Ants	Dowling and Oconnor, 2010
Hypoaspisella sp.1	A	Leaflitter soil	personal collection
Mysolaelaps sp.	E	Vertebrates	Dowling and Oconnor, 2010
Ololaelaps wangi	A	Decaying *Zea mags*; decaying *Phragmites communis* on the soil surface	Bai et al., 1996
Ondatralaelaps multispinosus	E	Musk rat (*Ondatra zibethica*)	Evans and Till, 1966

Ophionyssus natricis	E	Snakes and lizards	Evans and Till, 1966
Ornithonyssus bursa	E	*Hirundo rustica*	Masan et al., 2014
Ornithonyssus wernecki	E	*Didelphis albiventris*; *D. aurita*	Nieri-Bastos et al., 2011
Pseudoparasitus sp.	A	Living in nests of arthropods and vertibrates not parasitic	Dowling and Oconnor, 2010
Radfordiella oudemansi	E	*Desmodus rotundus rotundus*; *Diaemus youngi*	Radobsky, 1967
Steptolaelaps liomydis	E	*Liomys* spp.	Furman, 1955
Stratiolaelaps lamington	A	*Asplenium* litters, root ball, nest of rats,	Walter and Campbell, 2002
Stratiolaelaps miles	A	Dung pile, nest of *Microtus ochrogastor*	Walter and Campbell, 2002
Stratiolaelaps scimitus	A	*Rattus concolor* / *Rattus norvegicus*; Nest of muttonbirds; nest of wedge-tailed shearwater; nest of eclectus parrot	Womersley, 1954 / Walter and Campbell, 2002
Stratiolaelaps sp.1	A	Guano sediments in cave	personal collection
Tropilaelaps clareae	C	*Apis* spp.	Anderson and Morgan, 2007
Tropilaelaps koenigerum	C	*Apis* spp.	Anderson and Morgan, 2007
Tropilaelaps mercedesae	C	*Apis* spp.	Anderson and Morgan, 2007
Tropilaelaps thaii	C	*Apis* spp.	Anderson and Morgan, 2007
Varroa destructor	C	*Apis* spp.	Anderson and Trueman, 2000
Varroa jacobsoni	C	*Apis* spp.	Oudeman, 1904

***TABLE LEGNED.**

A. Free-living; B. Beetles; C. Bees; D. Ants; E. Vertebrates; F. Roaches; G. Diplopods; ?. not sure.
